# A Résumé of Twenty-five Cases of Abdominal Section

**Published:** 1883-01

**Authors:** J. Ewing Meaers

**Affiliations:** Surgeon to St. Mary’s Hospital, Demonstrator of Surgery in Jefferson Medical College, and Gynæcologist to Jefferson Medical College Hospital


A RÉSUMÉ OF TWENTY-FIVE CASES OF ABDOMINAL SECTION, By J. EWING MEAERS, M. D., Surgeon to St. Mary's Hospital, Demonstrator of Surgery in Jefferson Medical College, and Gynæcologist to Jefferson Medical College Hospital. [Read December 6, 1882.]

With a view of placing on record the results in a number of cases of abdominal section, and with the hope of contributing to the information possessed already with regard to these operations, I beg to submit the following *résumé* of the cases which have come under my care.

I have endeavored to present the points, which were regarded of interest, in a concise manner, for this purpose grouping them under different headings.

Of the twenty-five cases of abdominal section, twenty-two were performed for the removal of tumors from the ovary; one was made in a case of encysted dropsy of the peritoneum (reported in the Transactions of this College, 3d Series, Vol. I); one in a case of abdominal dropsy, in which the diagnosis was obscure, and an operation of exploration was made; and one for removal of the child in extra-uterine fœtation.

As the case of encysted dropsy has been reported to the College, and as I propose, at a future time, to report the one of extra-uterine pregnancy, I shall present on this occasion a *résumé* of the ovarian cases.

*Age of Patients.*—In the twenty-two cases the age varied from the youngest, sixteen, to the oldest, sixty-five years.

*Nationality.*—Sixteen patients were natives of the United States, and six of Ireland.

*Social Condition.*—Five were single, one was a widow, and sixteen were married.

*Duration of Growth.*—The duration of growth varied from three months to seven years, counting from the time at which the tumor was recognized first by the patient.

*Aspiration, or Previous Tapping.*—Aspiration, for the purpose of obtaining a specimen of fluid for examination, was performed in eight cases; tapping, to relieve abdominal distention, in four. In one case, in which the cyst was very fully distended by fluid, and the abdominal wall was very tense, leakage followed aspiration, and persisted for some hours, despite the efforts made to control it by pressure. In none of the cases, in which aspiration or tapping was performed, did any serious results occur, nor were there any evidences in the operations which followed, of complications due to the previous aspiration, or tapping. In all cases, proper precautions were taken, the patient being required to rest in bed from three to four days subsequent to the operation.

*Condition of the Patients at the Time of Operation.*—With the exception of two, who were the subjects of malignant disease of the ovary, the condition of the patients was good. All were placed upon preparatory treatment, extending over periods varying from two weeks to two months.

*Line of Incision.*—In all cases the abdominal cavity was opened by incision in the linea alba, midway between the umbilicus and pubes, the length of the incision varying according to the nature of the tumor and the presence or absence of adhesions. A simple, mono-cystic, non-adherent growth was extracted easily through an opening one and a half inches in length; whilst other tumors, polycystic in character, with numerous and strong adhesions required incisions from four to six inches in length in order that the hand could be introduced into the abdominal cavity, so as to sweep over the external surface of the tumor, for the purpose of detaching adhesions, and also into the interior of the growth to disintegrate its contents, and thus reduce its size. The incisions were invariably closed by the introduction of metallic sutures—iron or silver wire—the needle being carried so as to include the peritoneum.

*Adhesions.*—In eighteen cases adhesions, either parietal, omental, or visceral, existed—differing greatly as to extent and character. In some instances they were so slight as to be separated readily with the finger; in others, they were very extensive and very firm, requiring some force to effect their detachment, and exposing denuded, bleeding surfaces. In one of the fatal cases adhesions were universal, and so firm as to require a minute dissection to be made, in order to effect removal of the cyst.

In a second case a portion of the parietal surface of the peritoneum as large as the palm of the hand, and embracing the sub-peritoneal fascia, was detached, leaving a broad band of attachment. This was included in three animal ligatures, and the detached portion removed. During the period of recovery, which was not retarded, the patient referred to the position of the exposed surface as a sensitive point. In still another case, an adhesion, in the form of a cord, at least two inches in thickness, and from four to five inches in length, fastened the tumor to the parietes. It was drawn down and a double animal ligature applied before section was made. Hæmorrhage caused by the separation of adhesions has been controlled by the application of carbolized silk and animal ligatures, both ends being cut short, and the ligatures permitted to remain "*in situ.*" In some instances, torsion of the vessels has been sufficient to restrain the bleeding.

*Character of the Cysts.*—Four cysts were unilocular, sixteen were multilocular, and in two, malignant disease existed. In one of the latter medullary cancer involved both ovaries, and in the other colloid disease was present.

*Double ovariotomy* was performed in two cases.

*Primary or Secondary Operation.*—In one case the operation was secondary, ovariotomy having been performed thirteen years previously. In this instance the incision was made to the side of the cicatrix of the primary operation, so as to avoid wounding the pedicle of the tumor removed, which was found to exist as a small cord attached to the inner surface of the abdominal wall, and to be of such length as to permit the uterus to occupy a normal position. Elongation and shrinkage of the pedicle has been observed in post-mortem examinations made, in cases in which death occurred some years after ovariotomy.

*Treatment of the Pedicle.*—In all but one case the pedicle was secured by the application of the clamp; in the case excepted, a carbolized catgut ligature was applied, both ends cut short, and the pedicle was returned to the abdominal cavity. In cases of very short pedicles I have been able always to secure them with the clamp, and in no case was it observed that the traction made to accomplish this produced any harm. In one instance of an extremely short pedicle, where, in fact, the wall of the cyst was separated not more than a half inch from the uterus, a double animal ligature was applied, which failed to control the hæmorrhage. The clamp was then applied over the ligature, bringing the uterus well up between the edges of the incision. Although the patient had a tedious convalescence, the ligature and clamp came away in good time, and the abdominal incision healed kindly. Beneath the cicatrix the uterus could be distinctly outlined, and after the return of the patient to her usual duties no complaint was made of pain caused by traction upon the organ. In one case only was there noticed a slight tendency to the occurrence of ventral hernia, and this in a young patient who slipped and fell on the icy pavement a short time after recovery from the operation. A good deal of tension of the abdominal walls was felt in the act of falling, and it was thought a slight detachment of the pedicle had occurred. Rest in bed for a few days, with pressure over the cicatrix, relieved the condition.

In nearly all of the cases in which the clamp was applied it was observed that it could be removed safely at about the same time with the last of the sutures, and, therefore, the healing of the abdominal incision was not materially delayed. In one or two cases both sutures and clamp were permitted to remain longer than usual. In favorable cases the first of the sutures was usually removed on the sixth day, and the clamp on the eighth or tenth.

In three cases menstruation has occurred by the pedicle. In all of the cases it took place but once, and did not produce any serious inconvenience. It occurred in one of the cases in which double ovariotomy was performed, two clamps having been applied to the pedicles without difficulty.

Strangulation of the intestine has been noted as being due to to the use of the clamp. In my observation of the cases of others, and in my own, I have not met with an accident of this nature. As it has followed likewise the use of the ligature, it cannot be ascribed alone to the employment of the clamp.

The support afforded to the uterus by the attachment of the pedicle to the abdominal walls has seemed, in some of the cases, to have been of benefit, overcoming displacements which were productive of much previous discomfort.

In one of the three fatal cases which occurred, the ligature was applied and the pedicle returned to the abdominal cavity ; death resulted on the third day from septicæmia, and the autopsy showed the stump of the pedicle softened and covered by a grayish slough. This condition of the pedicle was, I think, not a primary condition, but was a part of the general inflammation which pervaded the abdominal cavity, occurring in a case in which the cyst was adherent in every part to the parietes and viscera, and which required minute dissection to effect its separation.

While the tendency of the present day is to return to the use of the ligature as an exclusive method of treating the pedicle, I think it unwise to discard entirely the clamp. The imbedding of the ligature and its subsequent absorption demand a degree of reparative power, which some much debilitated patients do not possess; in such cases it would appear proper to employ the clamp.

*Drainage.*—In one case it was thought desirable to secure drainage of the abdominal cavity after operation. For this purpose a large perforated rubber tube was introduced and allowed to remain in position for three days. During this period no fluid escaped, and the symptoms presented by the patient gave no indication of the collection of septic fluids. Of the great value of drainage after ovariotomy there can be no question. Its use is especially indicated in cases in which adhesions of some extent have existed.

*Antiseptic Precautions.*—In fourteen cases the antiseptic methods were employed in full detail at the time of operation, and partially during the conduct of the after-treatment, the spray being then omitted. The successful results which have attended some of the cases were undoubtedly due to its use. The condition of the patient during the after-treatment was favorably influenced, and convalescence was promoted. Of three fatal cases, one occurred after operation under the system. The tendency of most operators at the present time is to employ a modified form of the system, owing to the fear of constitutional impressions made by the agents employed. The constitutional effect of the carbolic acid has been observed in two or three cases in the condition of the urine ; other than this no symptoms were noted.

It has been stated above that menstruation by the pedicle occurred in three cases. In one a marked impression was made upon the temperature, and the elevation occuring as it did without being accompanied by a corresponding increase of the pulse-rate attracted attention. On the day preceding the appearance of the flow the pulse was 84 and the temperature normal, $98\frac{1}{2}°$. On the day of its appearance the temperature rose to 100°, and on the third day reached $100\frac{2}{5}°$, then declined to $99\frac{3}{5}$–99°, and on the day of the cessation of the flow returned to the normal, $98\frac{1}{2}$ —the pulse-rate in the meanwhile remained unchanged. As the elevation of the temperature occurred after convalescence had been fully declared and the patient was within two days of the period when she would have been permitted to sit up, some anxiety as to the cause existed which was not relieved until its relation to the presence of the menstrual flux was considered.

In two of the cases pregnancy occurred and terminated safely in connection with the development and growth of the cysts. In both, the cysts had attained large size, and notwithstanding the pressure exerted during parturition rupture did not occur. In one, puerperal peritonitis supervened, causing the formation of extensive adhesions, in the other, slight but firm adhesions were found.

In cases under my care recently, quinine has been administered in large doses in the twenty-four hours preceding the operation with a view to obviate shock, and in this respect its use has been attended with success. Thirty to sixty grains, in divided doses, have been given, and in each case so treated shock has been absent. In the preliminary and after-treatment it has also been given in tonic doses.

The duration of the operation has varied from thirty minutes to two hours, in the former time, mono-cystic non-adherent tumors have been removed and the wound closed. The latter period of time has been required to remove polycystic growths, with extensive and firm adhesions and many bleeding points to control. Serious complications during the operation and after-treatment have occurred in but two cases. In one already alluded to the adhesions were so extensive as to complicate seriously the operation and to render the result fatal. In the other, the slipping of the ligature and the persistence of hæmorrhage for some hours after the closure of the wound complicated the operation. This patient's recovery was slow, two months and a half elapsing before she was able to leave her bed, in which period there occurred in order the following complications—obstinate, uncontrollable diarrhœa, suppuration of hemorrhoids, formation of a large bed-sore over the region of the sacrum, with destruction of the sacro-coccygeal articulation and a condition of blood poisoning with swelling of the left parotid gland. Recovery finally took place, and the patient has been able to maintain herself by her work as a seamstress.

The size of the tumors varied greatly, and the weight from three to sixty pounds.

With one exception all the operations have been performed either in private houses or in a private hospital. One was operated upon in a private room of a general hospital, and in this a fatal result ensued—death, however, could not be attributed to this fact, but rather to the complications which existed in the case. In all cases careful attention was given to the preparation of the apartments, so that the patients should be placed under the most favorable hygienic conditions.

In the cases in which the progress was favorable the patients, as a rule, sat up in bed on the twelfth day, and on the fourteenth were permitted to get out and occupy the lounge or an easy chair. At the end of the third week gentle exercise about the house, and in favorable weather, in the open air was allowed. This exercise was continued daily, so as to prepare the patient, if living out of the city, for the journey home, which was undertaken between the fourth and fifth weeks after operation.

For twenty-four hours after the operation no food was given—at the end of this time, one ounce of milk, with lime-water, if vomiting had occurred, or if there was nausea, was given every three or four hours. In two or three days the amount of milk was increased to two ounces every three hours, alternating with a teaspoonful of beef-juice in three tablespoonsful of water. In some cases the beef-juice was administered instead of the milk from the first. As convalescence advanced additions were made cautiously to the diet, no solid food being given until the sutures and clamp had been removed and the bowels moved freely by enemata.

Usually enemata of soap water and olive oil were administered on the eighth day and on alternate days, subsequently, until evacuations occurred naturally. The catheter was used every six or eight hours for five days, and then efforts at evacuation of the bladder were permitted to be made by the patient.

When possible to avoid it, opiates were not administered. When required to relieve pain or secure rest, morphia in one-sixth to one-quarter of a grain was given hypodermically.

In the twenty-five abdominal sections death occurred in four cases—three after operation for the removal of ovarian cysts, and one after operation for the removal of the child in extra-uterine fœtation. Septicæmia was the cause of death in two of the ovarian operations, and in the case of extra-uterine pregnancy. Shock and hæmorrhage produced a fatal termination in the case of malignant disease of both ovaries in which double ovariotomy was performed.

